# Impact of sustained RNAi-mediated suppression of cellular cofactor Tat-SF1 on HIV-1 replication in CD4+ T cells

**DOI:** 10.1186/1743-422X-9-272

**Published:** 2012-11-15

**Authors:** Victoria A Green, Patrick Arbuthnot, Marc S Weinberg

**Affiliations:** 1 Antiviral Gene Therapy Research Unit, Health Sciences Faculty, University of the Witwatersrand, Johannesburg, South Africa; 2 Department of Molecular and Experimental Medicine, The Scripps Research Institute, La Jolla, CA, USA

## Abstract

**Background:**

Conventional anti-HIV drug regimens targeting viral enzymes are plagued by the emergence of drug resistance. There is interest in targeting HIV-dependency factors (HDFs), host proteins that the virus requires for replication, as drugs targeting their function may prove protective. Reporter cell lines provide a rapid and convenient method of identifying putative HDFs, but this approach may lead to misleading results and a failure to detect subtle detrimental effects on cells that result from HDF suppression. Thus, alternative methods for HDF validation are required. Cellular Tat-SF1 has long been ascribed a cofactor role in Tat-dependent transactivation of viral transcription elongation. Here we employ sustained RNAi-mediated suppression of Tat-SF1 to validate its requirement for HIV-1 replication in a CD4+ T cell-derived line and its potential as a therapeutic target.

**Results:**

shRNA-mediated suppression of Tat-SF1 reduced HIV-1 replication and infectious particle production from TZM-bl reporter cells. This effect was not a result of increased apoptosis, loss of cell viability or an immune response. To validate its requirement for HIV-1 replication in a more relevant cell line, CD4+ SupT1 cell populations were generated that stably expressed shRNAs. HIV-1 replication was significantly reduced for two weeks (~65%) in cells with depleted Tat-SF1, although the inhibition of viral replication was moderate when compared to SupT1 cells expressing a shRNA targeting the integration cofactor LEDGF/p75. Tat-SF1 suppression was attenuated over time, resulting from decreased shRNA guide strand expression, suggesting that there is a selective pressure to restore Tat-SF1 levels.

**Conclusions:**

This study validates Tat-SF1 as an HDF in CD4+ T cell-derived SupT1 cells. However, our findings also suggest that Tat-SF1 is not a critical cofactor required for virus replication and its suppression may affect cell growth. Therefore, this study demonstrates the importance of examining HIV-1 replication kinetics and cytotoxicity in cells with sustained HDF suppression to validate their therapeutic potential as targets.

## Background

Current anti-HIV drug regimens target several viral enzymes simultaneously, with the aim of preventing the emergence of drug resistance. However, efficacy of these drugs is limited by the problems of emergence of drug resistance that results from viral diversity and mutability. Host factors required by the virus for replication, so-called HIV-dependency factors (HDFs), represent attractive therapeutic targets since their coding sequences remain constant relative to the sequence variability of viral targets within a patient and across the pandemic.

Support for the notion that HDFs may be suitable therapeutic targets comes from a genome association study showing that single nucleotide polymorphisms in ZNRD1 are associated with slowed disease progression [1], and that a naturally occurring deletion in the CCR5 gene renders individuals resistant to an R5-tropic virus infection without associated physiological problems [2,3]. There have been several clinical trials showing the positive impact CCR5 deletion from CD4+ T cells has on T cell longevity, viral suppression and patient health (reviewed in [4]). This was most emphatically demonstrated by the apparent cure of the ‘Berlin patient’ [5-7]. There is therefore interest in identifying other HDFs that modulate HIV infection since drugs inhibiting their function may prove protective.

A number of reporter cell lines have been developed as convenient laboratory tools for the quantification of HIV replication. When coupled with RNA interference (RNAi)-mediated gene silencing, these models provide a rapid method for the identification of putative HDFs. This approach has been employed in genome-wide studies [8,9]. However, most putative HDFs identified by such approaches have yet to be validated in cells that are naturally infected by HIV. This is necessary as reporter cell lines may be misleading with respect to HDF importance, as exemplified in a study where only half of putative HDFs were validated as such in a T cell-derived line [10].

HIV-1 Tat-specific factor 1 (Tat-SF1) [NCBI RefSeq_peptide: NP_055315] has long been a candidate HDF since its identification as a cofactor for Tat-dependent transactivation of viral transcription elongation [11-14]. Tat-SF1 is an RNA-binding protein [12] that functions as a transcription elongation and splicing factor of cellular transcripts [15-17]. Most of the previous work on Tat-SF1 has focused on *in vitro* immunodepletion experiments of nuclear extracts. Other studies have demonstrated that RNAi-mediated suppression of Tat-SF1 inhibited HIV-1 replication in the HeLa-derived TZM-bl reporter cell line [8,18], mediated by a disruption to splicing of viral transcripts [18]. However, it was unknown whether this protein functions as an HDF in cells that are a natural target of HIV and, if so, whether the long-term impact of suppressing Tat-SF1 adversely affects these cells.

In this study we examined the impact of Tat-SF1 suppression, mediated by anti-Tat-SF1 short hairpin RNAs (shRNAs), in both TZM-bl reporter cells and CD4+ T cell-derived SupT1 cell lines. Inhibition of Tat-SF1 expression resulted in a significant inhibition of HIV-1 replication, although this was less pronounced than when suppressing the known lentiviral integration cofactor LEDGF/p75 [19,20]. In addition, Tat-SF1 suppression was attenuated during serial passage of transduced SupT1 cell lines, suggesting that Tat-SF1 suppression may confer a growth disadvantage to cells and therefore preclude its utility as a therapeutic target. The approach used here demonstrates that thorough analysis is required for HDF validation and detection of subtle changes to cell physiology that may result from HDF inhibition.

## Results

### RNAi-mediated suppression of Tat-SF1 without cytotoxicity

RNAi effectors, such as shRNAs, may be exploited to validate roles of HDFs. To suppress expression of endogenous Tat-SF1, which is encoded by the *HTATSF1* gene, three U6 RNA Polymerase (Pol) III shRNA expression cassettes, sh*htatsf1*-a, sh*htatsf1*-b and sh*htatsf1*-c, were generated (Additional file 1A). The shRNA loop sequences were derived from micro RNA- (miR-) 31. Through the introduction of mismatches in the anti-guide strand, G:U wobbles were created to enhance the thermodynamic asymmetry of the shRNA stems and facilitate intended mature guide strand bias [21-23].

**Figure 1:**
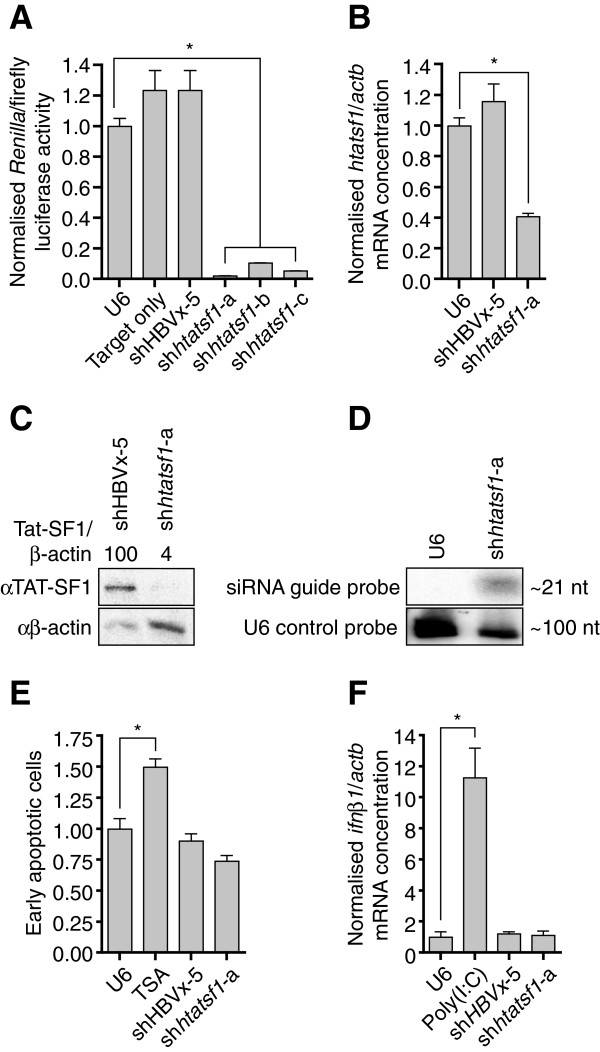
**shRNAs suppress Tat-SF1 expression without cytotoxicity.** 1**A**. Dual luciferase activities were assessed in HeLa cell lysates 48 h post-transfection with shRNA expression cassettes and psiCheck reporter constructs, in triplicate. Target *Renilla* luciferase levels are given relative to firefly luciferase and normalised to a mock construct with no shRNA expression (U6). shHBVx-5, which targets a sequence in HBV X protein, was included as a negative control. 1**B**. Total RNA was analysed by qRT-PCR 48 h post-transfection of TZM-bl cells with shRNA expression plasmids, or the U6 mock construct, in triplicate. Tat-SF1 mRNA (*htatsf1*) levels are given relative to β-actin mRNA (*actb*) normalised to the U6 control. 1**C**. TZM-bl cell lysates were subject to PAGE and Western blot 72 h post-transfection. Tat-SF1 expression is given relative to β-actin and normalised to the shHBVx-5 control. 1**D**. Total TZM-bl RNA isolated 48 h post-transfection was subject to small RNA PAGE and Northern blot. shRNA guide strand expression is given relative to U6 small nuclear RNA and normalised to the U6 control. 1**E**. TZM-bl cells were stained with Annexin V-conjugated FITC 72 h post-transfection, in duplicate. As a positive control for apoptosis induction, additional cells were treated with 500 nM trichostatin-A (TSA) 16 h pre-stain. Two images were acquired per sample and FITC levels quantified by ImageJ. 1**F**. Levels of interferon-β mRNA (*ifnb1*) relative to β-actin mRNA (*actb*) were determined by qRT-PCR on total cellular RNA extracted 48 h post-transfection, in triplicate. Poly(I:C) dsRNA was used as a positive control. Data are expressed as the mean ± SEM. *, *p* <0.05, one-way ANOVA with Dunnett post-tests relative to mock construct, U6.

Initial assessment of the ability of shRNAs to knockdown their cognate target sequences was made using a dual luciferase reporter assay. The three Tat-SF1 mRNA (*htatsf1*) target sites were inserted downstream of the *Renilla* luciferase ORF within a psiCheck dual-luciferase plasmid. Ratios of *Renilla* to constitutively expressed firefly luciferase activities were used to assess efficiency of shRNA-mediated target knockdown. All *htatsf1*-targeted shRNAs significantly reduced *Renilla*/firefly luciferase activity ratios compared to controls ie cells receiving the U6 plasmid, a construct with shRNA expression targeting hepatitis B virus X protein (shHBVx-5) [24] or the psiCheck target construct only (>90% knockdown; Figure 1A). Greatest knockdown was observed with sh*htatsf1*-a, which effectively inhibited expression of the endogenous mRNA target in TZM-bl cells, as determined by quantitative reverse transcription PCR (qRT-PCR) (~60% knockdown; Figure 1B). Western blot analysis demonstrated that sh*htatsf1*-a expression also mediated a significant reduction in Tat-SF1 (4% of shHBVx-5 control; Figure 1C). Small RNA Northern blot detected the ~21 nt sh*htatsf1*-a guide strand (Figure 1D), confirming that the exogenous shRNA was processed as intended and that the observed suppression of Tat-SF1 expression was mediated by an RNAi mechanism.

Ass essing the extent of toxic effects on introduction of shRNAs targeting Tat-SF1 expression is important, both in terms of validating this protein as a therapeutic target and in analysing the effect that the suppression of Tat-SF1 has on HIV-1 replication. Cytotoxicity may result from direct knockdown of Tat-SF1, non-specific silencing of cellular genes, or from induction of an innate immune response. The latter is likely to be triggered by the presence of exogenous double-stranded RNAs within the cell [25]. No increase in apoptosis was observed in TZM-bl cells 72 h post-sh*htatsf1*-a transfection, in contrast to cells treated with a high dose of the histone deacetylase inhibitor, trichostatin-A (Figure 1E). Neither was there altered mitochondrial dehydrogenase activity on sh*htatsf1*-a expression, compared with TZM-bl cells transfected with the U6 plasmid (Additional file 2). Induction of an innate immune response, monitored by quantification of interferon-β mRNA (*ifnb1*) expression, was also not evident (Figure 1F). Collectively these observations indicate that U6 RNA Pol III shRNA expression cassettes may be used to transiently silence Tat-SF1 expression without inducing apoptosis or an interferon response in TZM-bl cells.

### Suppression of Tat-SF1 inhibits HIV-1 replication in reporter cells

The effects of Tat-SF1 silencing on HIV-1 replication were initially assessed in TZM-bl cells. HeLa-derived TZM-bl cells may be infected with HIV-1 to a similar extent to human peripheral blood mononuclear cells (PBMCs) because they express transgenic HIV receptor CD4 and coreceptor CCR5 [26-28]. Furthermore, TZM-bl cells permit relatively simple assessment of HIV-1 replication as they contain an integrated Tat-dependent luciferase reporter [26-28].

**Figure 2:**
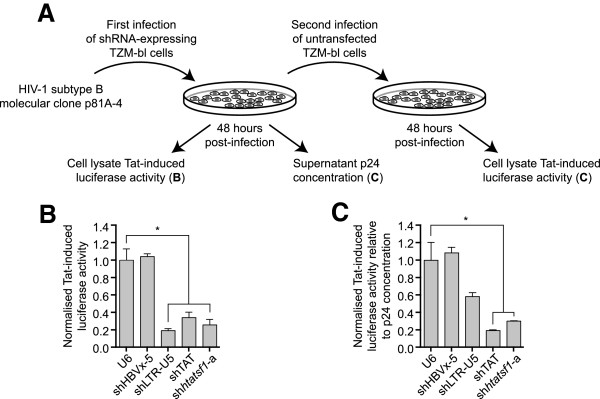
**Tat-SF1 suppression inhibits HIV-1 infectious particle production from TZM-bl cells.** 2**A**. Schematic of the HIV-1 infection protocol. 2**B**. TZM-bl cell lysates were analysed for luciferase activity 72 h post-transfection and 48 h post-infection with HIV-1^p81A-4^ at a TCID_50_ of 1000/ml, in triplicate. shLTR-U5 and shTAT, which target viral RNAs, were included as positive controls. 2**C**. Untransfected TZM-bl cell lysates were analysed for luciferase activity 48 h post-incubation with culture supernatant isolated from shRNA-expressing TZM-bl cells. Luciferase activity is given relative to culture supernatant p24 concentration, determined by ELISA. Data are expressed as the mean ± SEM. *, *p* <0.05, one-way ANOVA with Dunnett post-tests relative to mock construct, U6.

HIV-1 replication was quantified both by measurement of capsid protein p24 levels in culture supernatant and Tat-induced reporter gene activity (Figure 2A). Cells were transfected with the sh*htatsf1*-a expression construct, or controls, and infected 48 h later with virus derived from the HIV-1 subtype B molecular clone p81A-4 (HIV-1^p81A-4^) [29,30]. Tat-induced luciferase activity in cells with suppressed Tat-SF1 expression was ~20% of controls at 48 h after infection (Figure 2B). This effect was similar to that observed in cells expressing shTAT and shLTR-U5, previously developed shRNA expression cassettes that directly target sequences within the Tat open reading frame (ORF) and U5 region of the viral leader transcripts, respectively [31,32]. Tat-SF1 suppression also reduced infectious particle production by ~70% (Figure 2C). Collectively these results confirm previous reports that Tat-SF1 functions as an HDF in TZM-bl cells [8,18]. Given the limitations associated with transient host factor suppression for HDF validation, and the potential bias of reporter output, the impact of sustained Tat-SF1 suppression on HIV-1 replication kinetics over a time course was investigated.

### Stable expression of *htatsf1*-targeting shRNAs in SupT1 cells inhibits HIV-1 replication

The impact of sustained Tat-SF1 suppression on HIV-1 replication kinetics was assessed in CD4+ T cell-derived SupT1 cells [33], a model that more closely simulates natural HIV-1 infection than TZM-bl cells. An additional control shRNA was used, sh*psip1*-a, targeting the known HIV-1 cofactor LEDGF/p75 [20], which is encoded by the *PSIP1* gene. U6 RNA Pol III shRNA expression cassettes were incorporated into second-generation lentiviral vectors that also included a GFP reporter cassette. The dual luciferase reporter assay confirmed that the shRNAs remained capable of target silencing within the context of the lentivector (Additional file 3A). Recombinant lentiviruses were then generated and used to transduce SupT1 cells at a multiplicity of infection (MOI) of 0.15. After fluorescence activated cell sorting (FACS), a population of transduced SupT1 cells was propagated (Additional file 3B and C).

**Figure 3:**
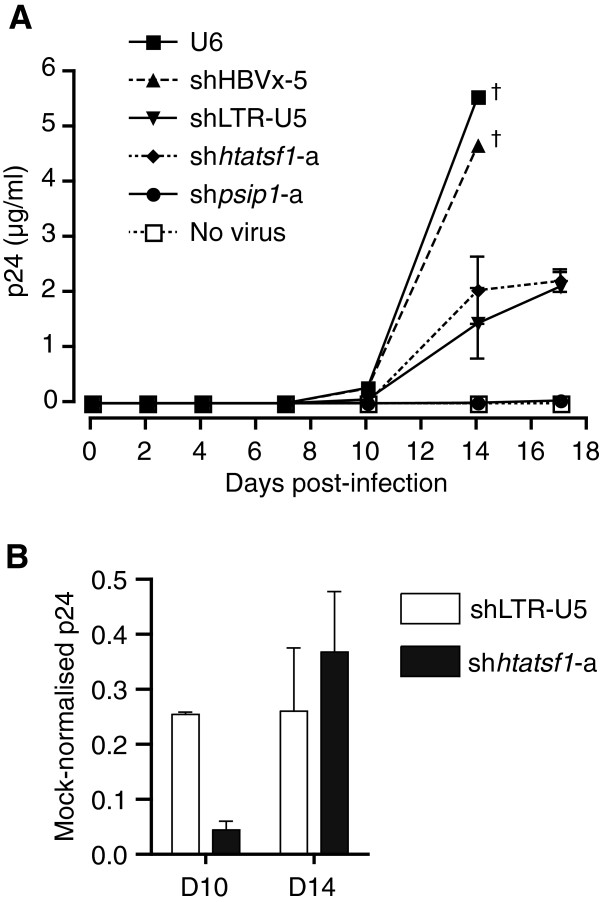
**Sustained Tat-SF1 suppression inhibits HIV-1 replication in CD4+ T cell-derived SupT1 cells.** SupT1 cell lines, with stable shRNA expression generated by lentiviral transduction, were infected with HIV-1^p81A-4^ at a TCID_50_ of 50/ml, in triplicate. 3**A**. Levels of p24 in culture supernatant were determined by ELISA 0, 2, 4, 7, 10, 14 and 17 days post-infection. Crosses indicate discontinued p24 measurement as a result of cell death. 3**B**. p24 levels in SupT1 cells expressing either shLTR-U5 or sh*htatsf1*-a, relative to the U6 mock, at days 10 and 14 post-infection. Data are expressed as the mean ± SEM.

SupT1 cells with stable shRNA expression were infected with HIV^p81A-4^. HIV-1 p24 concentrations in culture supernatant were measured regularly during a period of 17 days to assess HIV-1 replication kinetics (Figure 3A). The concentration of p24 rose to ~5 μg/ml on day 14 in the culture supernatant of control cells with no shRNA, or shHBVx-5, expression. No p24 measurement was made in these control cells on day 17 as a result of cell death from the high levels of virus replication. In contrast, p24 levels in culture supernatant of cells expressing sh*psip1*-a were only detected on day 4, and never reached more than 0.1 μg/ml during the time course, in accordance with the importance of LEDGF/p75 in HIV-1 replication [20]. Culture supernatant of cells with sh*htatsf1*-a expression exhibited p24 levels of ~2 μg/ml on day 14 (Figure 3A), a reduction of ~65% compared with the U6 mock, which was similar to that observed with shLTR-U5 expression (Figure 3B). These data show that sustained Tat-SF1 suppression inhibits HIV-1 subtype B replication in a T cell-derived line, albeit to a lesser extent than silencing of LEDGF/p75.

### Tat-SF1 expression increases following serial passage of sh*htatsf1*-a-expressing SupT1 cells

**Figure 4:**
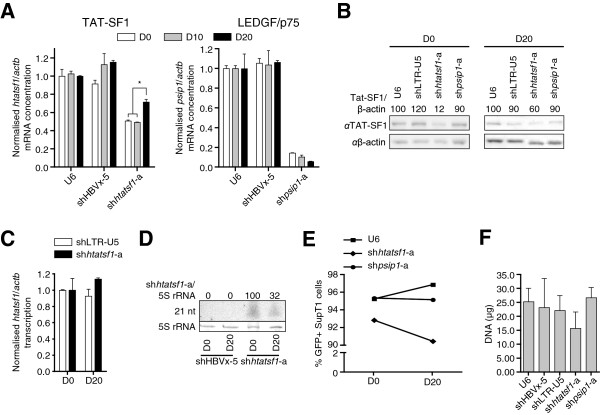
**Attenuation of shRNA-mediated Tat-SF1 suppression over time.** Samples were isolated from SupT1 cells with stable shRNA expression at time points equivalent to those of the HIV-1^p81A-4^ replication assay. 4**A**. Total SupT1 RNA was analysed by qRT-PCR, in triplicate. Target mRNA levels are given relative to β-actin mRNA (*actb*) normalised to the U6 cell line. Left panel: Tat-SF1 mRNA (*htatsf1*). Right panel: LEDGF/p75 mRNA (*psip1*). 4**B**. SupT1 cell lysates were subject to PAGE and Western blot. Day 20 samples were prepared in duplicate and representative blots are shown. Mean Tat-SF1 expression is given relative to β-actin and normalised to the U6 control at each time point. 4**C**. Nuclei isolated from SupT1 cells were subject to nuclear run-on analysis to quantify *htatsf1* transcription, in triplicate. Samples from both shLTR-U5- and sh*htatsf1*-a-expressing cells were normalised to those isolated at a time point equivalent to day 0 of the HIV-1^p81A-4^ replication assay. 4**D**. Total SupT1 RNA was subject to small RNA PAGE and Northern blot to assess sh*htatsf1*-a guide strand expression relative to 5S rRNAs. 4**E**. Proportion of GFP+ SupT1 cells. SupT1 cell populations were analysed by flow cytometry with 5 × 10^3^ events acquired per sample. 4**F**. shRNA-expressing SupT1 cell lines were cultured for 20 days prior to quantification of cellular DNA. Data are expressed as the mean ± SEM. *, *p* <0.05, two-way ANOVA with Bonferroni post-tests.

Close inspection of HIV^p81A-4^ replication kinetics reveals that on day 14, p24 levels in sh*htatfs1*-a‐expressing SupT1 cells, relative to the U6 control, were increased compared with day 10 (~95% versus ~65% knockdown; Figure 3B). In contrast, the suppression of p24 levels in shLTR-U5‐expressing cells was maintained at ~75%. The apparent attenuation of HIV-1 replication inhibition may result from adaptation of the virus to another cofactor, or may be a result of increased Tat-SF1 expression. However, cofactor adaptation is unlikely considering the duration of the assay. To determine whether there was increasing Tat-SF1 expression over the time course, SupT1 cell lines were raised and cultured for periods equivalent to the HIV^p81A-4^ replication assay. The level of *htatsf1* mRNA was suppressed throughout, compared to the U6 control, although *htatsf1* mRNA concentration increased significantly from day 10 (~49%) to day 20 (~70%; Figure 4A). These results were corroborated by Western blot analysis of Tat-SF1 expression (Figure 4B). In contrast, the degree of suppression of *psip1* mRNA was sustained in the sh*psip1*-a-expressing cell line throughout the time course (Figure 4A), demonstrating that the increase in shRNA target expression was specific to the sh*htatsf1*-a-expressing SupT1 cell line.

Several mechanisms, which are not mutually exclusive, may account for the observed increase in Tat-SF1 expression during serial passage of SupT1 cells expressing sh*htatsf1*-a. These are: (1) increased *HTATSF1* transcription; (2) reduced sh*htatsf1*-a expression; and, (3) positive selection for untransduced cells in the population where there is no Tat-SF1 suppression. Nuclear run-on analysis revealed no alteration in *HTATSF1* transcription rates, relative to transcription of *ACTB*, at day 20 compared with day 0 in SupT1 cells expressing sh*htatsf1*-a (Figure 4C). Northern blot analysis showed that expression of the sh*htatsf1*-a-derived guide strand was ~30% at day 20 of that detected on day 0 (Figure 4D), whereas the reduction in sh*psip1*-a-derived guide strand was less pronounced (~87% at day 20; Additional file 4). Flow cytometry on SupT1 cell lines over the time course showed that the GFP+ cells slightly diminished in the population transduced with sh*htatsf1*-a-expressing lentivirus, in contrast to SupT1 populations with no shRNA, or sh*psip1*-a, expression (Figure 4E). The size of the population transduced with sh*htatsf1*-a-expressing lentivirus was less than controls, as indicated by quantification of extracted DNA, although not significant (Figure 4F). Collectively, these data demonstrate that the inhibition of HIV-1 replication on Tat-SF1 suppression is attenuated over time as a result of an increase in Tat-SF1 expression. Such an increase is predominantly a result of a decrease in sh*htatsf1*-a guide strand expression.

## Discussion

Here we demonstrate that suppression of Tat-SF1 inhibits HIV-1 replication in both TZM-bl reporter cells and CD4+ T cell-derived SupT1 cells. Tat-SF1 has previously been shown to function as an HDF in TZM-bl cells [18], although we further demonstrated that the inhibitory effect on HIV-1 following RNAi-mediated Tat-SF1 suppression was not a result of cellular toxicity or induction of an immune response (Figure 1E and F) and includes inhibition of infectous particle production (Figure 2C). This study also examined the effect of sustained Tat-SF1 suppression on HIV-1 replication in T cell-derived SupT1 cells, a model that more closely mimics natural HIV-1 cellular targets. This approach had the added benefit of permitting quantification of HIV-1 replication kinetics for over two weeks. Tat-SF1 suppression inhibited HIV-1 replication in SupT1 cells throughout the time course (Figure 3A). Nevertheless, the inhibition of HIV-1 replication was modest compared with SupT1 cells with sustained suppression of the integration cofactor LEDGF/p75 (Figure 3A), suggesting that Tat-SF1 is a less critical HIV-1 cofactor than LEDGF/p75. This may be because Tat-SF1 is involved in increasing the efficiency of viral processes that still occur in its absence. This is consistent with its proposed function as one of a set of non-redundant RNA Pol II elongation factors that act cooperatively to facilitate efficient transcription elongation [16]. This is also consistent with observations that Tat-SF1 suppression results in a shift in the ratio of unspliced to spliced HIV-1 transcripts, but not complete loss of the spliced class [18]. These effects may be mediated by Tat-SF1 stabilising the large, multi-protein transcription elongation and splicing complexes [14], whilst not being critical for their activities. In contrast, our results confirm previous reports that LEDGF/p75 is a critical integration cofactor [20], and suggest that it is a good therapeutic target, as its suppression resulted in almost complete ablation of HIV-1 replication (Figure 3A). Indeed, there has been considerable progress in developing LEDGF/p75-HIV-1 integrase interaction inhibitors (reviewed in [34]).

Along with the limited inhibition of HIV-1 replication in SupT1 cells, other observations suggest that Tat-SF1 may not constitute a viable anti-HIV-1 therapeutic target. Tat-SF1 suppression was attenuated over time in SupT1 cells (Figure 4A and B) as a result, at least in part, of decreased sh*htatsf1*-a guide strand expression (Figure 4D). This may arise from epigenetic silencing of the shRNA expression cassette, or untransduced cells (or transduced cells with low or no sh*htatsf1*-a expression) within the population proliferating at a faster rate than those with sh*htatsf1*-a expression. Although these mechanisms are not mutually exclusive, our data favours the former as the primary mechanism for the reduction in guide strand expression, since the decrease in the percentage of GFP+ SupT1 cells is less than the reduction in sh*htatsf1*-a guide strand expression (Figure 4D and E). Regardless, when compared to the other SupT1 populations, the reduction in guide strand and percent of GFP+ cells was specific to the sh*htatsf1*-a population (Figure 4D and E, and Additional file 4), implying there is a selective pressure on cells to restore Tat-SF1 expression levels. Such a growth disadvantage on Tat-SF1 suppression would account for the small reduction in cell number within the population after serial passage (Figure 4F). This was not a significant difference, possibly because of adaptation to increase Tat-SF1 levels (Figure 4B).

Reduced Tat-SF1 expression may confer a growth disadvantage by disrupting expression of Tat-SF1 transcription and splicing targets, which have recently been shown to include genes involved in the cell cycle and nucleic acid metabolism [15]. Of course, reduced cell proliferation is not a desirable side effect, particularly in immune cells, which may preclude Tat-SF1 inhibition as an anti-HIV therapeutic strategy. However, it has been demonstrated that cells with greater resistance to HIV‐1 replication undergo preferential expansion *in vivo*[35]. Therefore, any growth disadvantage associated with Tat-SF1 suppression may be outweighed *in vivo* by a selective advantage in the context of an HIV-1 infection. Further experiments are needed to verify whether this is the case, but the observations reported here would certainly exclude prophylactic targeting of Tat-SF1. Nonetheless, as an HDF, Tat-SF1 expression heterogeneity should be considered a possible HIV-1 susceptibility factor.

More generally, this study highlights the limitations associated with HDF validation in a reporter cell line. Although convenient, there may be bias toward host factors with a more direct influence on reporter gene expression. In addition, the expression levels of host factors differ between cell types, which may alter HIV-1 replication kinetics [36], particularly in reporter cell lines that are not derived from natural targets of HIV-1. Furthermore, measurement of TZM-bl reporter gene activity requires cell lysis, preventing serial monitoring of HIV-1 replication and, as such, are most useful for transient suppression experiments, which may lead to overlooking HDFs with long half-lives and not detect subtle detrimental effects on cell physiology resulting from HDF suppression. Thus the limitations of bias, cell type and transient suppression that are associated with reporter cell lines may cause a distortion in the relative importance and therapeutic potential of an HDF. This was observed in this study, where transient suppression-experiments in TZM-bl cells suggested that Tat-SF1 was a critical HIV-1 cofactor, in contrast to the findings from sustained suppression-experiments in SupT1 cells, an approach which is less subject to distortion from bias and cell type. Furthermore, this study reveals that Tat-SF1 suppression may confer a growth disadvantage only apparent on serial passage of cells. In contrast, previous reports that LEDGF/p75 comprises a promising therapeutic target were confirmed. Overall this study provides an experimental template for the approach required to validate HDFs and the therapeutic potential of their targeting, and should be extended to putative HDFs identified by genome-wide screens.

## Conclusions

HDFs represent potential therapeutic targets and, as such, putative HDFs require validation. Focusing on the HIV-1 cofactor Tat-SF1, this study highlights the limitations associated with HDF validation in the TZM-bl reporter cell line. We demonstrate an alternative approach for determining the impact that host factor suppression has on HIV-1 replication and cell physiology, which employs sustained RNAi-mediated host factor suppression in a cell line derived from a physiological substrate of HIV-1. This approach was used to validate Tat-SF1 as an HDF in CD4+ T cell-derived SupT1 cells: sustained RNAi-mediated Tat-SF1 suppression inhibits HIV-1 replication in SupT1 cells. However, the inhibitory effect was modest compared to cells with sustained suppression of the integration cofactor LEDGF/p75, suggesting that Tat-SF1 is not a critical HIV-1 cofactor. Furthermore, Tat-SF1 suppression is attenuated over time, suggesting that reduced Tat-SF1 levels confer a growth disadvantage to cells. Thus, whilst this study reveals that Tat-SF1 functions as an HDF in SupT1 cells, further studies are required to determine whether variants might modulate HIV-1 infection and its suppression would have a long-term inhibitory effect on HIV-1 replication *in vivo*.

## Methods

### shRNA constructs

shRNAs sh*htatsf1*-a, sh*htatsf1*-b and sh*htatsf1*-c were designed to target *htatsf1* transcript [NCBI RefSeq_RNA: NM_014500.3] at the sequences GCT ACA TAT CAG GCC AAT TAT, GCG CAT CTA GTT CTA CCG CAA and CTG CAA CTG GAA TGG CGT T, respectively (Additional file 1). These target sites were selected from sequences suggested by The RNAi Consortium ( http://www.broad.mit.edu/genome_bio/trc/rnai.html). All shRNAs were designed to contain a loop sequence derived from miR-31. G:U mismatches were incorporated at the 3’ end of the anti-guide strand of some shRNAs to decrease thermodynamic stability of this end of the hairpin stem and favour selection of the intended guide strand. RNA Pol III U6 shRNA expression cassettes were generated by a two-step PCR approach described previously [37]. These were cloned into pTZ57R/T (Fermentas). Construct sequence was confirmed by automated cycle sequencing.

Several previously developed constructs were used as controls in experiments: a mock pTZU6+1 (U6) construct with no shRNA sequence [38]; a shRNA negative control, shHBVx-5, which targets an irrelevant site in hepatitis B virus (HBV) X protein [24]; and, two positive controls, shLTR-U5 and shTAT, which are named after the location of their target sequences within HIV-1 transcripts and were initially developed based on subtype B molecular clone HXB2 [GenBank: K03455] [31,32]. shRNA sh*psip1*-a was adapted from a guide strand previously shown to inhibit LEDGF/p75 expression [20] that targets the p75 isoform of *psip1* transcript [NCBI RefSeq_RNA: NM_033222.2] at the sequence GAC AGC ATG AGG AAG CGA A.

### Cell culture and transfections

HeLa-derived TZM-bl cells (NIH AIDS Research and Reference Reagent Program), which express the HIV receptor CD4 and coreceptor CCR5 and contain a luciferase reporter driven by a Tat-inducible LTR promoter derived from pSG3.1 [GenBank: L02317] [26-28], were maintained in Dulbecco’s Modified Eagle’s Medium (DMEM), supplemented with 10% heat-inactivated fetal calf serum (FCS), at 37°C and 5% CO_2_. HEK293T, HeLa and SupT1 cells (NIH AIDS Research and Reference Reagent Program), the latter a non-Hodgkin’s T cell lymphoma suspension cell line expressing high levels of surface CD4 [33], were maintained in the same media.

Transfections were carried out using 1 μl Lipofectamine2000 (Invitrogen) to 1 μg DNA, according to manufacturer’s instructions. Medium was changed 5 h post-transfection. Where appropriate, a plasmid with constitutive eGFP expression (pCI-eGFP) was cotransfected followed by fluorescence microscopy 48 h later to verify equivalent transfection efficiencies [39].

### Dual luciferase reporter assay

To generate psiCheck target plasmids, with all shRNA target sites for each cellular factor adjacent to one another, complementary oligonucleotides were treated with polynucleotide kinase (Promega), annealed and cloned directly into the *Xho*I-*Not*I sites of psiCheck2. An *Eco*RV site was inserted within each annealed dsDNA insert to facilitate screening. The oligonucleotides used for psiCheck *htatsf1* were: TCG AGA TAT CGC TAC ATA TCA GGC CAA TTA TGC GCA TCT AGT TCT ACC GCA AAC TGC AAC TGG AAT GGC GTT GC; and, CTA GAT GCG CAT AAT TGG CCT GAT ATG TAG CGA TAT CGG CCG CAA CGC CAT TCC AGT TGC AGT TTG CGG TAG AA; and, for psiCheck *psip1* were: TCG AGA TAT CAG ACA GCA TGA GGA AGC GAA GCA GCT ACA GAA GTC AAG ATT GC; and, GGC CGC AAT CTT GAC TTC TGT AGC TGC TTC GCT TCC TCA TGC TGT CTG ATA TC. Target constructs psiCheck HBVx [40] and psiCheck LTR [31] have been described previously.

HeLa or HEK293T cells were seeded at 5.0 × 10^4^ and 1.2 × 10^5^ cells per well, respectively, in a 24-well culture plate and transfected 24 h later with 500 ng shRNA expression construct, 100 ng of psiCheck target reporter construct and 10 ng pCI-eGFP, in triplicate. Firefly and *Renilla* luciferase activities were determined 48 h later using the Dual Luciferase Reporter Assay System (Promega) and a Veritas dual-injection luminometer (Turner Biosystems), according to manufacturer’s instructions. *Renilla*: firefly luciferase activity ratios were normalised to the U6 control mean.

### Quantitative RT-PCR of cellular factor mRNAs

TZM-bl cells were seeded at 5.0 × 10^4^ cells per well in a 24-well culture plate and transfected 24 h later with 500 ng shRNA expression construct and 10 ng pCI-eGFP, in triplicate. Total TZM-bl cellular RNA was extracted using TriReagent (Sigma-Aldrich) 48 h later, or from stably transduced SupT1 cells cultured for periods equivalent to days 0, 10 and 20 of the HIV-1 replication assay (see below). Total RNA was subjected to DNase treatment (Promega) and random-primed reverse-transcription using the SuperScript III reverse transcriptase (RT) (Invitrogen). cDNA was analysed for target mRNA expression relative to β-actin mRNA (*actb*) transcript NM_01101.2 using the SensiMix *Lite* Kit (Quantace) with the following primers: *htatsf1* forward AGTGGGACCTGGACAAAAAGG; *htatsf1* reverse GTT CCG GGG CTT TTT CTT GTG; *psip1* forward GCT GAA CAA AGA CAG CAT GAG GA; *psip1* reverse ATT GCT CTC CCC GTT ATG TTG TG; *actb* forward AGG TCA TCA CCA TTG GCA ATG AG; and, *actb* reverse TCT TTG CGG ATG TCC ACG TCA. The qPCR was performed in a Carousel-based Lightcycler V.2 System (Roche) with the following parameters: denaturation at 95°C for 10 min, 50 cycles of denaturation at 95°C, annealing at 60°C and extension at 72°C, each for 10 s. Amplification cycles were followed by melting curve analysis to verify the specificity of the PCR products. No RT controls were included for each sample and no cDNA controls for each primer set. Target mRNA: *actb* ratios were normalised to the mean expression ratio of U6-transfected samples.

### Western blot

TZM-bl cells were seeded at 1.5 × 10^5^ cells per well in a 6-well culture plate and transfected 24 h later with 2 μg shRNA expression construct and 10 ng pCI-eGFP. Cells were harvested 72 h post-transfection and lysed with RIPA buffer. Total protein was quantified using the BCA Protein Assay Kit (Pierce). A ladder composed of IgG-binding proteins ranging from 22 to 120 kDa in size and 80 μg of samples were resolved on a 12% polyacrylamide gel. Protein was transferred to a PVDF membrane (Millipore) and probed with rabbit polyclonal antibodies to Tat-SF1 (a gift from M. Garcia-Blanco) at 1:100 and β-actin (GenWay Biotech) at 1:1,000. The latter was used to quantify loading of samples. HRP-conjugated donkey anti-rabbit IgG secondary antibody (GenWay Biotech) was used at a dilution of 1:25,000 and proteins were detected with SuperSignal West Pico Chemiluminescent Substrate (Pierce). Images were acquired with a G-BOX (Syngene). Levels of target protein are reported relative to levels of β-actin and normalised to the shHBVx-5 control.

SupT1 cells were similarly analysed by Western blot, with the exception that cells were harvested after culture periods equivalent to days 0 and 20 of the HIV-1 replication assay (see below). Day 20 samples were prepared in duplicate. Mean target protein expression relative to levels of β-actin are reported normalised to the U6 mock at each time point.

### Northern blot analysis of shRNA guide strand processing

TZM-bl cells were seeded at 2 × 10^6^ cells in a 60 cm^2^ culture dish and transfected with 20 μg shRNA expression plasmid 24 h later. Total cellular RNA was isolated from TZM-bl cells 48 h post-transfection, or SupT1 cells, using TriReagent (Sigma-Aldrich). Thirty micrograms of RNA was resolved on urea denaturing 15% polyacrylamide gels and blotted onto nylon membranes. RNA molecular weight markers were run alongside the cellular RNA. Blots were hybridised to DNA oligonucleotide probes of complementary sequence to hairpin-derived guide strands and, therefore, of the same sequence as the shRNA target sequences (see above).

For analysis of TZM-bl RNA, the RNA ladder and DNA probes were labelled at their 5’ ends with [γ-^32^P] ATP and T4 polynucleotide kinase. To quantify loading of the TZM-bl RNA, an oligonucleotide sequence complementary to U6 small nuclear RNA was used of the following sequence: TAG TAT ATG TGC TGC CGA AGC GAG CA. Following hybridisation, blots were exposed to an imaging plate and viewed on a FLA-7000 phosphorimager (Fujifilm), stripped and reprobed.

For SupT1 RNA analysis, levels of 5S rRNAs on the ethidium bromide-stained polyacrylamide gel verified equal loading of the samples. The RNA ladder and DNA probes were labelled at their 3’ ends with the DIG Oligonucleotide 3’-end Labelling Kit according to manufacturer’s instructions (Roche). Following hybridisation, chemiluminescence detection of bound probes was enabled by incubation of the membranes with alkaline phosphatase-conjugated anti-DIG antibody, incubation with CDP-*Star* (Roche) and image acquisition with a G-BOX (Syngene).

### Apoptosis quantification

TZM-bl cells were seeded at 3 × 10^4^ cells per well on CELLocate microgrid coverslips (Eppendorf) in a 24-well culture plate. Cells were transfected with 500 ng shRNA expression constructs 24 h later, in duplicate. Another subset of cells was treated with 500 nM trichostatin-A 80 h post-seeding as a positive control. Seventy-two hours post-transfection, or 16 h post-trichostatin-A treatment, apoptosis was quantified using the TACS Annexin V-FITC Apoptosis Detection Kit (R&D Systems). Fluorescence images were acquired for two fields of view per well on an Axiovert 100 M microscope with image capture by AxioVision 2.0.5 software (Carl Zeiss Microimaging). Fluorescence was quantified using ImageJ 1.40 g (developed by W. Rasband, NIH) and reported normalised to the U6 mock.

### MTT assay for cell viability

TZM-bl cells were seeded at 1 × 10^4^ cells per well in a 96-well culture plate. Cells were either transfected with 100 ng shRNA expression construct, or treated with 10, 100 or 500 nM trichostatin-A, 24 h later, in triplicate. A further 48 h later, 0.1 mg of 3-(4,5-dimethylthiazol-2-yl)-2,5-diphenyltretrazolium bromide (MTT) was added to each well. Cells were incubated at 37°C for 1 h, media removed and formazan precipitates resuspended in 200 μl DMSO. Absorbance at 570 nm, with a reference wavelength of 655 nm, was determined in a Model 680 microplate reader (BioRad) and reported normalised to the cell control, which was not transfected or treated with TSA.

### Immune response

TZM-bl cells were seeded at 3 × 10^4^ cells per well in a 24-well culture plate and transfected with 500 ng shRNA expression construct or 1 μg of the double-stranded RNA polyinosinic:polycytidylic acid (poly(I:C) (Sigma-Aldrich) as a positive control, in triplicate. Total RNA was extracted using TriReagent (Sigma-Sldrich) 48 h post-transfection and subject to DNase treatment, reverse transcription and qPCR, as described above. Primers used to amplify interferon-β mRNA (*ifnb1*) were: forward TCC AAA TTG CTC TCC TGT TGT GCT; and, reverse CCA CAG GAG CTT CTG ACA CTG AAA A. *ifnb1*:*actb* expression ratios were normalised to the mean expression ratio of U6-transfected samples.

### Virus preparation and propagation

1.2 × 10^6^ HEK293T cells were seeded in a 25 cm^2^ culture flask and transfected 24 h later, using PolyFect transfection reagent (Qiagen), with 4 μg of subtype B molecular clone p81A-4 (HIV-1^p81A-4^) (NIH AIDS Research & Reference Reagent Program) [29,30]. Media was replaced 24 h later. A further 24 h later, media was collected, filtered, made up to 20% FCS, aliquoted and stored at −80°C.

Median tissue culture infectious dose (TCID_50_) was determined using the Spearman-Karber method [41,42]. TZM-bl and SupT1 cells were seeded at 1 × 10^4^ cells per well in a 96-well culture plate and infected with various dilutions of virus, in triplicate, 24 h later. For TZM-bl cells, infections were carried out in the presence of 15 μg/ml DEAE-D. Cells were washed with PBS 24 h post-infection, referred to as day 0. For TZM-bl cells, luciferase activities were determined in cell lysates 48 h post-infection using the Bright-Glo Luciferase Assay System (Promega). Samples were considered luciferase positive if the luminescence signal was greater than that of the mean of the no virus samples plus two standard deviations. SupT1 cells were incubated for 7 days post-washing and both day 0 and day 7 culture supernatant samples were analysed for the HIV-1 antigen p24 by ELISA using the HIV antigen mAb Kit (Murex Biotech). Samples were classed as positive if the A_450_ was greater than the absorbance of the kit’s negative control + 0.50.

### HIV-1 replication in TZM-bl reporter cells

TZM-bl cells were seeded at 5 × 10^4^ cells per well in a 24-well culture plate and transfected 24 h later with 500 ng shRNA expression constructs and 10 ng pCI-eGFP, in triplicate. Cells were infected with either FV5 or HIV-1^p81A-4^ at a TCID_50_ of 1000/ml 24 h later in the presence of 15 μg/ml DEAE-D. Cells were washed with PBS 24 h post-infection. Forty-eight hours post-infection, 100 μl of culture supernatant was removed and stored at −80°C for subsequent analysis of p24 levels using the HIV antigen mAb Kit (Murex Biotech). Another 100 μl of culture supernatant was used to infect additional TZM-bl cells, seeded at 5 x 10^4^ cells per well in a 24-well culture plate the preceeding day, in the presence of 15 μg/ml DEAE-D. Tat-induced luciferase activities were determined in cell lysates 48 h post-infections using the Bright-Glo Luciferase Assay System (Promega). Data are reported normalised to the U6 mock.

### Generation of shRNA-expressing SupT1 cell lines

shRNA expression cassettes were excised from pTZ plasmids by digestion with *Eco*RI and *Acc*I and cloned into the *Eco*RI and *Cla*I sites of second generation lentivector pLVTH (Addgene plasmid 12262, deposited by D. Trono) [43], which encodes a GFP reporter. Lentiviruses were generated from the shRNA-expressing lentivectors by transfecting 3.6 × 10^6^ HEK293T cells in a 60 cm^2^ culture dish 24 h later with 5 μg shRNA-expressing lentivector, 3.8 μg psPAX2 and 2.5 μg pMD2.G (Addgene plasmids 12260 and 12259, respectively, both deposited by D. Trono). Culture media collected 24 and 48 h post-transfection was pooled, filtered and stored at −80°C. Lentiviruses were titred based on non-linear regression of the number of GFP+ SupT1 cells following transduction with various dilutions of lentivirus. This was determined using a FACSCalibur flow cytometer (BD Biosciences) to acquire 5 × 10^3^ events per sample with analysis by FlowJo 9.1 (Tree Star). SupT1 cells were gated based on forward and side scatter characteristics and GFP+ cells determined from that subset by comparison of transduced with untransduced cells.

SupT1 cells were seeded at 3 × 10^5^ cells per 75 cm^2^ culture flask and incubated with lentivirus at a MOI of 0.15. Cells were cultured for 5 days prior to harvest and fluorescence activated cell sorting (FACS) on a FACSCalibur. Sorted GFP+ cells were concentrated by centrifugation and cultured in DMEM with 20% FCS, 100 U/ml penicillin, 100 μg.ml streptomycin, 50 μg/ml tetracycline, 100 μg/ml ampicillin, 170 μg/ml chloramphenicol, 50 μg/ml kanamycin and 100 μg/ml ciprofloxacin for 1 week. Sorted cell lines were cultured for a further week without antibiotics and stocks made. The proportion of GFP+ SupT1 cells in each cell line was determined by flow cytometry and FlowJo analysis (Tree Star) based on the acquisition of 5 × 10^3^ events immediately prior to sorting (pre-sort) and freezing (post-sort). Thawed SupT1 cell lines were cultured for 5 days prior to seeding in all subsequent experiments.

### HIV-1^p81A-4^ replication in SupT1 cell lines

SupT1 cell lines with shRNA expression were seeded at 2 × 10^4^ cells per well in a round-bottomed 96-well culture plate and immediately infected with HIV-1^p81A-4^ at a TCID_50_ of 50/ml in duplicate. Mock SupT1 cells with the U6 promoter but no shRNA expression were cultured both with and without infection as controls. Twenty-four hours post-infection, cells were washed with PBS, resuspended in 350 μl media and pelleted prior to removal of 150 μl media as day 0 samples. Cells were resuspended with replacement of the media removed. Cells were pelleted and another 150 μl media sample removed seventy-two hours post-infection (day 2). Samples were removed in the same fashion on days 4, 7, 10, 14 and 17. All samples were stored at −80°C prior to analysis of p24 content by ELISA (Murex Biotech). Dilutions of the kit positive control were used to generate a standard curve of p24 levels from which absolute levels of p24 in the experimental samples were determined.

### Nuclear run-on analysis of *htatsf1* transcription

SupT1 cell lines expressing either sh*htatsf1*-a or shLTR-U5 were cultured for periods equivalent to days 0 and 20 of the HIV-1^p81A-4^ replication assay before harvesting of cell nuclei, in triplicate. Nuclear run-on was performed as previously described [44], with modification to use biotin-tagged transcripts [45]. Biotinylated RNA was isolated using Dynabeads MyOne Streptavidin C1 beads (Invitrogen), prior to reverse transcription and qPCR. *htatsf1*:*actb* transcription ratios were normalised to the mean expression ratio of day 0 samples.

### Proliferation of SupT1 cell lines

SupT1 cell lines were analysed by flow cytometry after culture for periods equivalent to days 0 and 20 of the HIV-1^p81A-4^ replication assay (see below). The proportion of GFP+ SupT1 cells in each population was determined following acquisition of 5 × 10^3^ events on a FACSCalibur (BD Biosciences) and analysis using FlowJo 9.1 (Tree Star). SupT1 cell lines with shRNA expression were also seeded at 5 × 10^4^ cells per well in a 12-well plate in quadruplicate. After 20 days culture, cellular DNA was extracted and quantified by NanoDrop (Thermo Fisher Scientific), in duplicate.

### Statistics

Data are expressed as the mean ± the standard error of the mean (SEM). Statistical difference was considered significant (*) when *p* <0.05. Data were analysed using non-linear regression, unpaired *t*-test, one-way ANOVA, followed by Dunnett’s multiple comparison post-tests, and two-way ANOVA, followed by Bonferroni post-tests, where appropriate, using Prism 4.0c (GraphPad Software).

## Competing interests

The authors declare that no competing interests exist.

## Authors' contributions

VAG conceived, designed and performed the experiments, analysed the data and wrote the paper. PA and MSW conceived experiments and wrote the paper. All authors read and approved the final manuscript.

## Supplementary Material

Additional file 1 **Tat-SF1-targeting shRNAs.** Schematic of predicted structures of shRNAs targeting Tat-SF1 mRNA (htatsf1**).** G:U wobble base-pairs, through the introduction of mismatches in the anti-guide strand, are indicated by black triangles. Click here for file

Additional file 2 **shRNA expression does not alter cell viability.** TZM-bl cells were treated with MTT 48 h post-transfection with shRNA expression cassettes. Trichostatin-A (TSA) was used as a positive control for reduced cell viability. Mitochondrial dehydrogenase activity is reported normalised to the cell control that was untransfected and untreated with TSA. Data are expressed as the mean ± SEM. *, *p* <0.05, one-way ANOVA with Dunnett post-tests relative to mock construct, U6. Click here for file

Additional file 3 **Generation of shRNA-expressing SupT1 cell lines.** S3A. Dual luciferase activities were assessed in HEK293T cell lysates 48 h post-transfection with lentivector shRNA expression cassettes and cognate psiCheck reporter constructs, in triplicate. Target *Renilla* luciferase levels are given relative to firefly luciferase and normalised to the U6 mock construct for each psiCheck reporter. Data are expressed as the mean ± SEM. *, *p* <0.05, two-way ANOVA with Bonferroni post-tests. S6B. Representative flow cytometry plots of the SupT1 cell sorting strategy. SupT1 cells were transduced with lentivirus carrying shRNA expression constructs and a GFP reporter at a MOI of 0.15. These populations were sorted to generate a population with >90% GFP expression for use in all subsequent experiments. S6C. Proportion of GFP+ SupT1 cells in each population pre- and post-sort based on acquisition of 5 × 10^3^ events by flow cytometry. Click here for file

Additional file 4 **Time course of sh*psip1*-a guide strand expression in SupT1 cells.** Total SupT1 RNA was subject to small RNA PAGE and Northern blot to assess sh*psip1*-a guide strand expression relative to 5S rRNAs. Samples were isolated at time points equivalent to days 0 and 20 of the HIV-1^p81A-4^ replication assay. Click here for file

## References

[B1] Fellay J Shianna KV Ge D Colombo S Ledergerber B Weale M Zhang K Gumbs C Castagna A Cossarizza A Cozzi-Lepri A De Luca A Easterbrook P Francioli P Mallal S Martinez-Picado J Miro JM Obel N Smith JP Wyniger J Descombes P Antonarakis SE Letvin NL McMichael AJ Haynes BF Telenti A Goldstein DB A whole-genome association study of major determinants for host control of HIV-1 Science 2007 317 944 947 10.1126/science.1143767 17641165 PMC1991296

[B2] Huang Y Paxton WA Wolinsky SM Neumann AU Zhang L He T Kang S Ceradini D Jin Z Yazdanbakhsh K Kunstman K Erickson D Dragon E Landau NR Phair J Ho DD Koup RA The role of a mutant CCR5 allele in HIV-1 transmission and disease progression Nat Med 1996 2 1240 1243 10.1038/nm1196-1240 8898752

[B3] Liu R Paxton WA Choe S Ceradini D Martin SR Horuk R MacDonald ME Stuhlmann H Koup RA Landau NR Homozygous defect in HIV-1 coreceptor accounts for resistance of some multiply-exposed individuals to HIV-1 infection Cell 1996 86 367 377 10.1016/S0092-8674(00)80110-5 8756719

[B4] Cohen J The emerging race to cure HIV infections Science 2011 332 784 785 787–789 10.1126/science.332.6031.784 21566173

[B5] Allers K Hutter G Hofmann J Loddenkemper C Rieger K Thiel E Schneider T Evidence for the cure of HIV infection by CCR5{Delta}32/{Delta}32 stem cell transplantation Blood 2011 117 10 2791 2799 10.1182/blood-2010-09-309591 21148083

[B6] Hutter G Ganepola S Eradication of HIV by transplantation of CCR5-deficient hematopoietic stem cells ScientificWorldJournal 2011 11 1068 1076 21552772 10.1100/tsw.2011.102 PMC5720062

[B7] Hutter G Nowak D Mossner M Ganepola S Mussig A Allers K Schneider T Hofmann J Kucherer C Blau O Blau IW Hofmann WK Thiel E Long-term control of HIV by CCR5 Delta32/Delta32 stem-cell transplantation N Engl J Med 2009 360 692 698 10.1056/NEJMoa0802905 19213682

[B8] Brass AL Dykxhoorn DM Benita Y Yan N Engelman A Xavier RJ Lieberman J Elledge SJ Identification of host proteins required for HIV infection through a functional genomic screen Science 2008 319 921 926 10.1126/science.1152725 18187620

[B9] Zhou H Xu M Huang Q Gates AT Zhang XD Castle JC Stec E Ferrer M Strulovici B Hazuda DJ Espeseth AS Genome-scale RNAi screen for host factors required for HIV replication Cell Host Microbe 2008 4 495 504 10.1016/j.chom.2008.10.004 18976975

[B10] Eekels JJ Geerts D Jeeninga RE Berkhout B Long-term inhibition of HIV-1 replication with RNA interference against cellular co-factors Antiviral Res 2011 89 43 53 10.1016/j.antiviral.2010.11.005 21093490

[B11] Parada CA Roeder RG A novel RNA polymerase II-containing complex potentiates Tat-enhanced HIV-1 transcription EMBO J 1999 18 3688 3701 10.1093/emboj/18.13.3688 10393184 PMC1171446

[B12] Zhou Q Sharp PA Tat-SF1: cofactor for stimulation of transcriptional elongation by HIV-1 Tat Science 1996 274 605 610 10.1126/science.274.5287.605 8849451

[B13] Zhou M Deng L Lacoste V Park HU Pumfery A Kashanchi F Brady JN Kumar A Coordination of transcription factor phosphorylation and histone methylation by the P-TEFb kinase during human immunodeficiency virus type 1 transcription J Virol 2004 78 13522 13533 10.1128/JVI.78.24.13522-13533.2004 15564463 PMC533906

[B14] Kim JB Yamaguchi Y Wada T Handa H Sharp PA Tat-SF1 protein associates with RAP30 and human SPT5 proteins Mol Cell Biol 1999 19 5960 5968 10454543 10.1128/mcb.19.9.5960 PMC84462

[B15] Miller HB Robinson TJ Gordan R Hartemink AJ Garcia-Blanco MA Identification of Tat-SF1 cellular targets by exon array analysis reveals dual roles in transcription and splicing RNA 2011 17 665 674 10.1261/rna.2462011 21282347 PMC3062177

[B16] Chen Y Yamaguchi Y Tsugeno Y Yamamoto J Yamada T Nakamura M Hisatake K Handa H DSIF, the Paf1 complex, and Tat-SF1 have nonredundant, cooperative roles in RNA polymerase II elongation Genes Dev 2009 23 2765 2777 10.1101/gad.1834709 19952111 PMC2788331

[B17] Li XY Green MR The HIV-1 Tat cellular coactivator Tat-SF1 is a general transcription elongation factor Genes Dev 1998 12 2992 2996 10.1101/gad.12.19.2992 9765201 PMC317190

[B18] Miller HB Saunders KO Tomaras GD Garcia-Blanco MA Tat-SF1 is not required for Tat transactivation but does regulate the relative levels of unspliced and spliced HIV-1 RNAs PLoS One 2009 4 e5710 10.1371/journal.pone.0005710 19479034 PMC2682658

[B19] Llano M Vanegas M Fregoso O Saenz D Chung S Peretz M Poeschla EM LEDGF/p75 determines cellular trafficking of diverse lentiviral but not murine oncoretroviral integrase proteins and is a component of functional lentiviral preintegration complexes J Virol 2004 78 9524 9537 10.1128/JVI.78.17.9524-9537.2004 15308744 PMC506940

[B20] Llano M Saenz DT Meehan A Wongthida P Peretz M Walker WH Teo W Poeschla EM An essential role for LEDGF/p75 in HIV integration Science 2006 314 461 464 10.1126/science.1132319 16959972

[B21] Ma JB Yuan YR Meister G Pei Y Tuschl T Patel DJ Structural basis for 5'-end-specific recognition of guide RNA by the A. fulgidus Piwi protein Nature 2005 434 666 670 10.1038/nature03514 15800629 PMC4694588

[B22] Parker JS Roe SM Barford D Structural insights into mRNA recognition from a PIWI domain-siRNA guide complex Nature 2005 434 663 666 10.1038/nature03462 15800628 PMC2938470

[B23] Schwarz DS Hutvagner G Du T Xu Z Aronin N Zamore PD Asymmetry in the assembly of the RNAi enzyme complex Cell 2003 115 199 208 10.1016/S0092-8674(03)00759-1 14567917

[B24] Carmona S Ely A Crowther C Moolla N Salazar FH Marion PL Ferry N Weinberg MS Arbuthnot P Effective inhibition of HBV replication in vivo by anti-HBx short hairpin RNAs Mol Ther 2006 13 411 421 10.1016/j.ymthe.2005.10.013 16337206

[B25] Karpala AJ Doran TJ Bean AG Immune responses to dsRNA: implications for gene silencing technologies Immunol Cell Biol 2005 83 211 216 10.1111/j.1440-1711.2005.01331.x 15877597

[B26] Derdeyn CA Decker JM Sfakianos JN Wu X O'Brien WA Ratner L Kappes JC Shaw GM Hunter E Sensitivity of human immunodeficiency virus type 1 to the fusion inhibitor T-20 is modulated by coreceptor specificity defined by the V3 loop of gp120 J Virol 2000 74 8358 8367 10.1128/JVI.74.18.8358-8367.2000 10954535 PMC116346

[B27] Platt EJ Wehrly K Kuhmann SE Chesebro B Kabat D Effects of CCR5 and CD4 cell surface concentrations on infections by macrophagetropic isolates of human immunodeficiency virus type 1 J Virol 1998 72 2855 2864 9525605 10.1128/jvi.72.4.2855-2864.1998 PMC109730

[B28] Wei X Decker JM Liu H Zhang Z Arani RB Kilby JM Saag MS Wu X Shaw GM Kappes JC Emergence of resistant human immunodeficiency virus type 1 in patients receiving fusion inhibitor (T-20) monotherapy Antimicrob Agents Chemother 2002 46 1896 1905 10.1128/AAC.46.6.1896-1905.2002 12019106 PMC127242

[B29] Chesebro B Wehrly K Nishio J Perryman S Macrophage-tropic human immunodeficiency virus isolates from different patients exhibit unusual V3 envelope sequence homogeneity in comparison with T-cell-tropic isolates: definition of critical amino acids involved in cell tropism J Virol 1992 66 6547 6554 1404602 10.1128/jvi.66.11.6547-6554.1992 PMC240149

[B30] Walter BL Wehrly K Swanstrom R Platt E Kabat D Chesebro B Role of low CD4 levels in the influence of human immunodeficiency virus type 1 envelope V1 and V2 regions on entry and spread in macrophages J Virol 2005 79 4828 4837 10.1128/JVI.79.8.4828-4837.2005 15795268 PMC1069537

[B31] Barichievy S Saayman S von Eije KJ Morris KV Arbuthnot P Weinberg MS The inhibitory efficacy of RNA POL III-expressed long hairpin RNAs targeted to untranslated regions of the HIV-1 5' long terminal repeat Oligonucleotides 2007 17 419 431 10.1089/oli.2007.0095 17896874

[B32] Saayman S Barichievy S Capovilla A Morris KV Arbuthnot P Weinberg MS The efficacy of generating three independent anti-HIV-1 siRNAs from a single U6 RNA Pol III-expressed long hairpin RNA PLoS One 2008 3 e2602 10.1371/journal.pone.0002602 18596982 PMC2434202

[B33] Smith SD Shatsky M Cohen PS Warnke R Link MP Glader BE Monoclonal antibody and enzymatic profiles of human malignant T-lymphoid cells and derived cell lines Cancer Res 1984 44 5657 5660 6437672

[B34] De Luca L Ferro S Morreale F Chimirri A Inhibition of the interaction between HIV-1 integrase and its cofactor LEDGF/p75: a promising approach in anti-retroviral therapy Mini Rev Med Chem 2011 11 714 727 21651465 10.2174/138955711796268787

[B35] Swan CH Buhler B Steinberger P Tschan MP Barbas CF 3rd Torbett BE T-cell protection and enrichment through lentiviral CCR5 intrabody gene delivery Gene Ther 2006 13 1480 1492 10.1038/sj.gt.3302801 16738691

[B36] Li J Liu Y Kim T Min R Zhang Z Gene expression variability within and between human populations and implications toward disease susceptibility PLoS Comput Biol 2010 26 8 6 10.1371/journal.pcbi.1000910 PMC2928754 20865155

[B37] Castanotto D Li H Rossi JJ Functional siRNA expression from transfected PCR products RNA 2002 8 1454 1460 10.1017/S1355838202021362 12458798 PMC1370351

[B38] Bertrand E Castanotto D Zhou C Carbonnelle C Lee NS Good P Chatterjee S Grange T Pictet R Kohn D Engelke D Rossi JJ The expression cassette determines the functional activity of ribozymes in mammalian cells by controlling their intracellular localization RNA 1997 3 75 88 8990401 PMC1369464

[B39] Passman M Weinberg M Kew M Arbuthnot P In situ demonstration of inhibitory effects of hammerhead ribozymes that are targeted to the hepatitis Bx sequence in cultured cells Biochem Biophys Res Commun 2000 268 728 733 10.1006/bbrc.2000.2209 10679273

[B40] Weinberg MS Ely A Barichievy S Crowther C Mufamadi S Carmona S Arbuthnot P Specific inhibition of HBV replication in vitro and in vivo with expressed long hairpin RNA Mol Ther 2007 15 534 541 10.1038/sj.mt.6300077 17213835

[B41] Chou TC Talalay P Quantitative analysis of dose-effect relationships: the combined effects of multiple drugs or enzyme inhibitors Adv Enzyme Regul 1984 22 27 55 6382953 10.1016/0065-2571(84)90007-4

[B42] Kahan BD Gibbons S Tejpal N Chou TC Stepkowski S Synergistic effect of the rapamycin-cyclosporine combination: median effect analysis of in vitro immune performances by human T lymphocytes in PHA, CD3, and MLR proliferative and cytotoxicity assays Transplant Proc 1991 23 1090 1091 1824888

[B43] Wiznerowicz M Trono D Conditional suppression of cellular genes: lentivirus vector-mediated drug-inducible RNA interference J Virol 2003 77 8957 8961 10.1128/JVI.77.16.8957-8951.2003 12885912 PMC167245

[B44] Morris KV Chan SW Jacobsen SE Looney DJ Small interfering RNA-induced transcriptional gene silencing in human cells Science 2004 305 1289 1292 10.1126/science.1101372 15297624

[B45] Zhang MX Ou H Shen YH Wang J Coselli J Wang XL Regulation of endothelial nitric oxide synthase by small RNA Proc Natl Acad Sci U S A 2005 102 16967 16972 10.1073/pnas.0503853102 16284254 PMC1287968

