# Supplementary figures and images for: Impact of sustained RNAi-mediated suppression of cellular cofactor Tat-SF1 on HIV-1 replication in CD4+ T cells
Source: Virol J. 2012 Nov 15;9:272. doi: 10.1186/1743-422X-9-272 (PMC3511259; doi:10.1186/1743-422X-9-272)

*shhtatsf1-a*

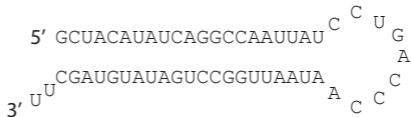

*shhtatsf1-b*

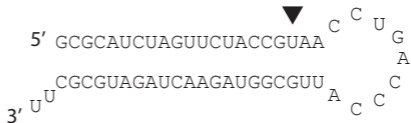

*shhtatsf1-c*

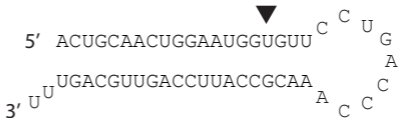

Supplement: Additional file 1 — Tat-SF1-targeting shRNAs. Schematic of predicted structures of shRNAs targeting Tat-SF1 mRNA (htatsf1). G:U wobble base-pairs, through the introduction of mismatches in the anti-guide strand, are indicated by black triangles. [file 1743-422X-9-272-S1.pdf]

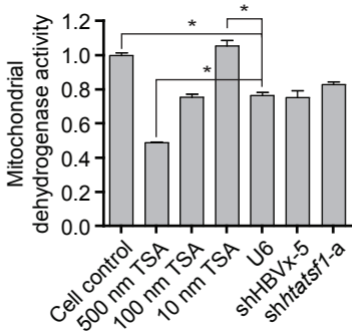

Supplement: Additional file 2 — shRNA expression does not alter cell viability. TZM-bl cells were treated with MTT 48 h post-transfection with shRNA expression cassettes. Trichostatin-A (TSA) was used as a positive control for reduced cell viability. Mitochondrial dehydrogenase activity is reported normalised to the cell control that was untransfected and untreated with TSA. Data are expressed as the mean ± SEM. *, p <0.05, one-way ANOVA with Dunnett post-tests relative to mock construct, U6. [file 1743-422X-9-272-S2.pdf]

**A**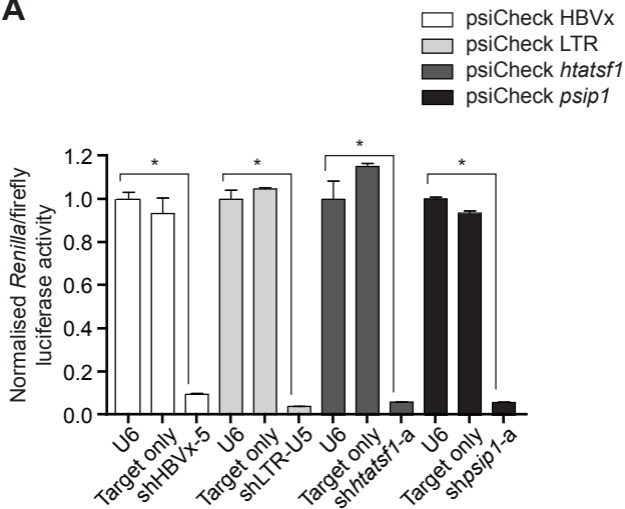**B**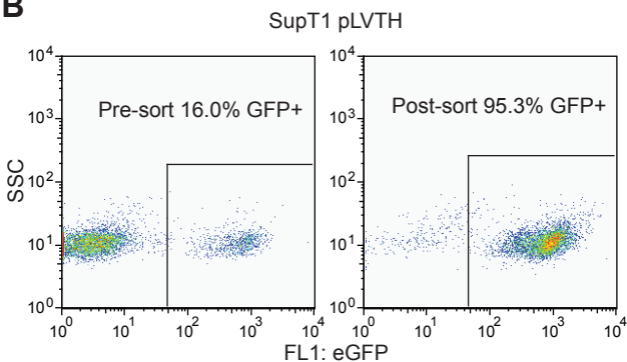**C**

| cell line            | % GFP    |           |
|----------------------|----------|-----------|
|                      | pre-sort | post-sort |
| U6                   | 16.0     | 95.3      |
| shHBVx-5             | 15.2     | 93.6      |
| shLTR-U5             | 16.9     | 91.9      |
| sh <i>htatsf1</i> -a | 17.7     | 92.9      |
| sh <i>psip1</i> -a   | 18.0     | 95.4      |

Supplement: Additional file 3 — Generation of shRNA-expressing SupT1 cell lines. S3A. Dual luciferase activities were assessed in HEK293T cell lysates 48 h post-transfection with lentivector shRNA expression cassettes and cognate psiCheck reporter constructs, in triplicate. Target Renilla luciferase levels are given relative to firefly luciferase and normalised to the U6 mock construct for each psiCheck reporter. Data are expressed as the mean ± SEM. *, p <0.05, two-way ANOVA with Bonferroni post-tests. S6B. Representative flow cytometry plots of the SupT1 cell sorting strategy. SupT1 cells were transduced with lentivirus carrying shRNA expression constructs and a GFP reporter at a MOI of 0.15. These populations were sorted to generate a population with >90% GFP expression for use in all subsequent experiments. S6C. Proportion of GFP+ SupT1 cells in each population pre- and post-sort based on acquisition of 5 × 10^3^ events by flow cytometry. [file 1743-422X-9-272-S3.pdf]

*shpsip1-a/*

5S rRNA

0

0

100

87

21 nt

5S rRNA

D0

D20

D0

D20

*shHBVx-5*

*shpsip1-a*

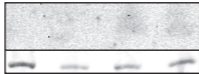

Supplement: Additional file 4 — Time course of shpsip1-a guide strand expression in SupT1 cells. Total SupT1 RNA was subject to small RNA PAGE and Northern blot to assess shpsip1-a guide strand expression relative to 5S rRNAs. Samples were isolated at time points equivalent to days 0 and 20 of the HIV-1p81A-4 replication assay. [file 1743-422X-9-272-S4.pdf]
